# A Self-Supported CuO/Cu Nanowire Electrode as Highly Efficient Sensor for COD Measurement

**DOI:** 10.3390/molecules24173132

**Published:** 2019-08-28

**Authors:** Xinwen Huang, Yingying Zhu, Wanquan Yang, Anhua Jiang, Xiaoqiang Jin, Yirong Zhang, Liang Yan, Geshan Zhang, Zongjian Liu

**Affiliations:** 1 Institute of Environment, College of Chemical Engineering, Zhejiang University of Technology, Hangzhou 300014, China; 2 Powerchina Huadong Engineering Corporation Limited, Hangzhou 311122, China; 3 College of Chemical Engineering, Zhejiang University of Technology, Hangzhou 300014, China

**Keywords:** Chemical oxygen demand, Copper oxide nanowires, Cyclic voltammogram, Electrochemical sensor

## Abstract

A self-supported CuO/Cu nanowire electrode (CuO/CuNWE), which was prepared by annealing Cu nanowires to form a porous Cu nanowire electrode (CuNWE) and then anodizing the as-prepared CuNWE in alkaline medium to generate Cu(OH)_2_ nanowires followed by calcination, was employed for chemical oxygen demand (COD) determination using cyclic voltammetry (CV). The structure and electrochemical behavior of the CuO/CuNWE were investigated by scanning electron microscopy, X-ray diffraction, and CV. The results indicated that the as-synthesized CuO/CuNWE, in which CuO nanowires with a length of several micrometers and a diameter of 100 to 300 nm could be found, was stable in alkaline medium and more electrocatalytically active for oxidizing a wide range of organic compounds in comparison with the CuNWE. Under optimized alkaline concentration and scan rate, the CuO/CuNWE exhibited a good performance for COD measurement, with a linear range of 5 to 1153 mg L^−1^, a sensitivity of 2.46× 10^−2^ mA /(mg L^−1^), and a detection limit of about 2.3 mg L^−1^. In addition, an excellent correlation was observed in COD values obtained by our method and the classic dichromate method (r = 0.9995, *p* < 0.01, n = 11). Finally, our method was successfully used to measure the COD values in real water samples, showing great potential for practical application in water pollution control.

## 1. Introduction

Chemical oxygen demand (COD), an important indicator in environmental monitoring, is widely employed to evaluate the amount of organic compounds in wastewater and surface water [1,2]. The standard method for COD determination is defined as a measurement of the amount of oxygen equivalently consumed in the oxidation of organic compounds to carbon dioxide, ammonia, and water by strong oxidants under acidic conditions, as in the case of dichromate titration [3]. Despite its widespread use, this standard method involves several inherent disadvantages, such as low detection sensitivity, low time efficiency, complicated operation, existence of incomplete oxidation, non-recycling, consumption of expensive (e.g., Ag_2_SO_4_), highly corrosive (e.g., H_2_SO_4_) and/or toxic (e.g., Cr_2_O_7_^2−^) chemicals, and the problem of secondary pollution [4]. Therefore, the development of simple, rapid, sensitive, cost-effective, and environmentally friendly methods for COD measurement has always been a sustained priority.

Electrochemical techniques have exhibited great potential as an alternative approach for COD measurement because they are highly sensitive, efficient, inexpensive, and conveniently handling [5]. In order to fulfill the direct detection of COD, many materials have been tested as electrocatalysts, including CoO [6], Cu [7,8,9,10,11], Ni-Cu [12], TiO_2_ [13,14], PbO_2_ [15], nano-Pt [16], CuO/AgO [17], Cu-Co [18], nickel nanoparticle/nafion-graphene oxide (GO) [19], boron doped diamond(BDD) [20,21], Ti/Sb–SnO_2_/PbO_2_ [22], and carbon fiber felt/CeO_2_-β-PbO_2_ [23]. Among them, Cu-base materials have been recognized as promising electrocatalysts for determination of COD in water samples due to their strong ability to electro-oxidize organic compounds [24] and have drawn increasing attention in the past [7,8,9,10,11,12]. For example, Silva et al. used a Cu rod electrode as the sensor and found that it exhibited a wide COD linear range (53.0–2801.4 mg/L) but a high detection limit (20.3 mg/L) despite having a good ability to produce active species CuO(OH) [7]. Compared to the Cu rod electrode, Cu or CuO nanomaterials modified electrodes, such as nano-Cu/copper-disk [8], nano-Cu/glassy carbon electrode (GCE) [9], and nano-Cu/Cu-cable [10] have been found to have a higher detection sensitivity and a lower detection limit because Cu or CuO nanomaterials own a large specific surface area and thus a high electrocatalytic activity. However, the substrates used, e.g., GCE and Cu rod or foil, often possessing a low specific surface area, and thus cannot provide enough room for decoration of Cu or CuO nanoparticles. Recently, we have found that thermal annealing of Cu nanowires (CuNWs) can produce a self-supported porous Cu nanowire electrode (CuNWE) with high surface area [25,26,27]. We feel that use of the CuNWE as substrate for decoration or growth of CuO should be a good choice for generating a CuO/Cu sensor for COD detection because a porous CuNWE is not only more highly conductive compared to GCE, but also has a higher specific surface area than both GCE and Cu rod or foil.

Based on the above considerations, in this article we present an investigation of a self-supported CuO/Cu nanowire electrode (CuO/CuNWE) as COD sensor. The CuO/CuNWE was prepared by annealing Cu nanowires to form a porous CuNWE and then anodizing the as-prepared CuNWE in alkaline medium to generate Cu(OH)_2_ nanowires, followed by calcination. The in situ growth of CuO nanowires via electrochemical method can avoid the use of polymer binder (e.g., nafion), which may reduce the charge transfer resistance, and thus improve performance of the as-synthesized sensor [28]. The structure and electrochemical behavior of the CuO/CuNWE were characterized by scanning electron microscopy (SEM), X-ray diffraction (XRD), and cyclic voltammetry (CV). The CuO/CuNWE-based electrochemical sensor was used to determine the COD values of real water samples collected from industries and surface waters. 

## 2. Results and Discussion

### 2.1. Characterizations of CuNWE and CuO/CuNWE 

**Figure 1:**
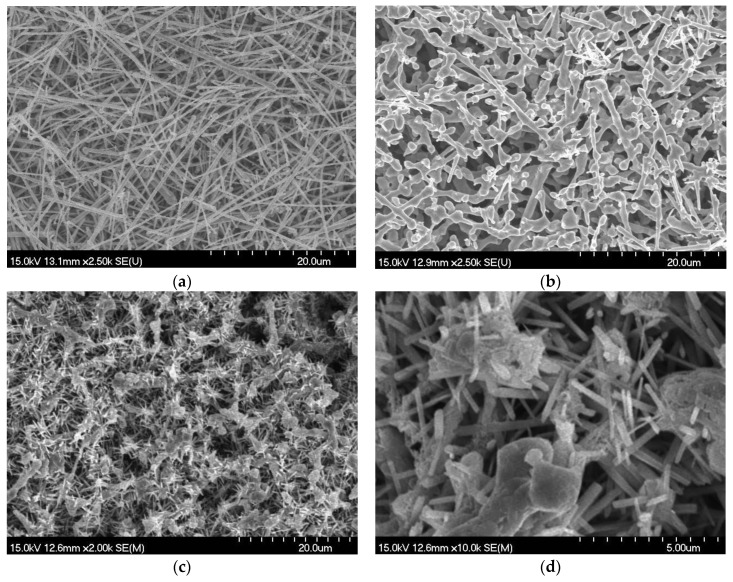
SEM images of (**a**) CuNW film before annealing, (**b**) CuNWE, (**c**) and (**d**) CuO/CuNWE.

The detailed preparation process of the CuNWE can be found in the Materials and Methods section. According to our previous study, the calcination of CuNWs at 600 °C can generate the self-supported CuNWE with good physical stability [26]. By following a similar preparation procedure, the morphology of the resulting CuNWE in this study is similar to that in our previous research [26]. Before annealing (Figure 1a), the diameter of the nanowires ranges from tens of nanometers to hundreds of nanometers. After calcination at 600 °C for 30 min (Figure 1b), nanowires grow thicker and interlace with each other to form a porous network. Such a porous structure can offer a much higher electrochemical active surface area than that of a Cu wire electrode having nearly the same geometrical area as the CuNWE [26]. In order to prepare a self-supported CuO/CuNWE electrode that can benefit from the high electrical conductivity of Cu, the grown CuO nanowires shouldn’t be too long, so that the porous CuNW substrate should not be heavily destroyed by anodization (Note: CuO nanowires that are too long will also lead to a high charge transfer resistance because of the low electrical conductivity of CuO). In our pilot study we found that when the NaOH concentration was fixed at 3 M (an alkaline concentration used in anodizing Cu foil by Li et al. [28]) and the anodization duration was set at 5 min, too high (e.g., ≥1.5 V) or too low (e.g., ≤1.1 V) voltage used in the anodization cannot lead to the formation of nanowires on the CuNWE (see Table S1 and Figure S1 in Supplementary Materials). Despite that nanowires can be formed at a voltage of 1.3 V after 5 min of anodization (Figure S1 in Supplementary Materials), the length of the nanowire would be too long to achieve a fast charge transfer. Therefore, we reduce the anodization duration to 1 min to prepare CuO/CuNWE. The morphology of the CuO/CuNWE is presented in Figure 1c. It is clear that many wire-like materials can be found on the surface of the electrode. A magnification view of the CuO/CuNWE indicates that these wire-like materials have a diameter in the range of about 100 to 300 nm and a length of several micrometers (Figure 1d). Compared to the CuNWE, growth of these nanowires is expected to enhance the surface area and thus provide more active sites for catalysis. 

**Figure 2:**
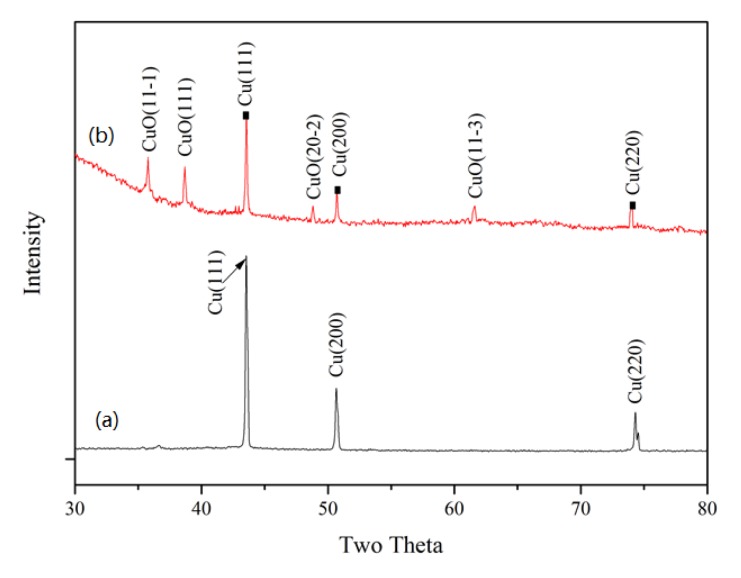
XRD patterns of (**a**) CuNWE and (**b**) CuO/CuNWE.

To identify the phase composition of these nanowires, the CuO/CuNWE was characterized by XRD. In comparison to the XRD patterns of the CuNWE (Figure 2a) where the diffraction peaks at 43.2°, 50.3°, and 74.0° can be ascribed to Cu with a face-centered cubic structure (JCPDS 04-0836), new diffraction peaks positioned at 35.5°, 38.7°, 48.7°, and 61.5° appear in the XRD patterns of the CuO/CuNWE. These diffraction peaks can be indexed to cuprous oxide (JCPDS 48-1548) and correspond to the diffractions of the crystal planes of (11-1), (111), (20-2), and (11-3), respectively. These results confirm that the newly grown nanowires should be CuO, and the as-prepared CuO/CuNWE is made up of both Cu and CuO. 

### 2.2. Electrochemical Behavior of CuNWE and CuO/CuNWE in Alkaline Medium

Although the electrochemistry of copper and copper-based electrodes in alkaline solutions has been extensively investigated [29,30,31,32,33,34], the exact mechanism for oxidation of organic materials on these electrodes in alkaline medium is still in dispute. Three types of active species for the oxidation of organics have been proposed, including Cu(III) species [30], radicals (e.g., hydroxyl radicals [30] or CuOOH**⋅** [7]), and surface-bonded copper (I) hydrous oxide species [Cu^+^⋅ nH_2_O]_ads_ [32]. The most accepted mechanism was proposed by Marioli and Kuwana [30], where Cu(III) species, e.g., CuOOH or Cu_2_O_3_, were treated as active species for oxidizing organics. The oxidization of organic compounds by Cu(III) species involves a redox mediation electron transfer process with hybrid-steps [34], which can be described as below [31]:

Cu+ 2OH^−^ → Cu(OH)_2_ + 2e^−^ (1)

Cu(OH)_2_ + OH^−^ → Cu(III)OOH +H_2_O + e^−^ (2)

CuO+ OH^−^ → Cu(III)OOH + e^−^ (3)

CuO + H_2_O + 2OH^−^ → Cu(OH)_4_^−^+ e^−^ (4)

Cu(III)OOH + Organics(red) + H_2_O → Cu(OH)_2_ + Organics(ox) + OH^−^ (5)

#### 2.2.1. Electrochemical Behavior in Organics-Absent Alkaline Medium

**Figure 3:**
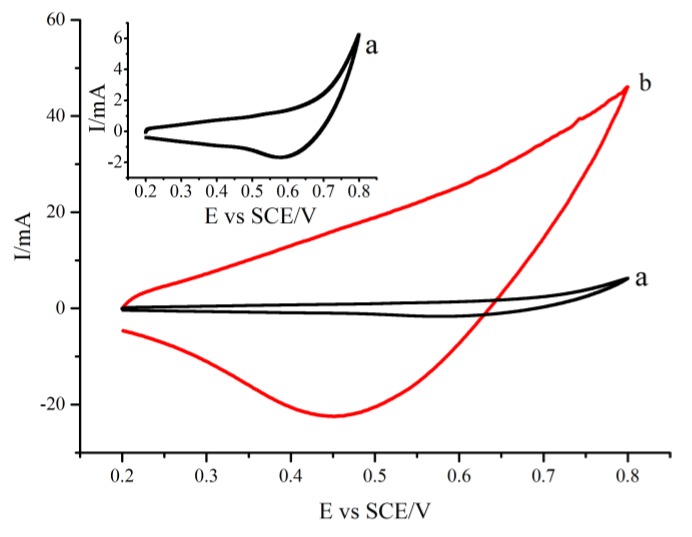
CV curve recorded at the CuNWE (a) and CuO/CuNWE (b) in a 0.075 M NaOH solution at a scan rate of 0.05 V s^−1^. Inset is the magnified view of curve a.

**Figure 4:**
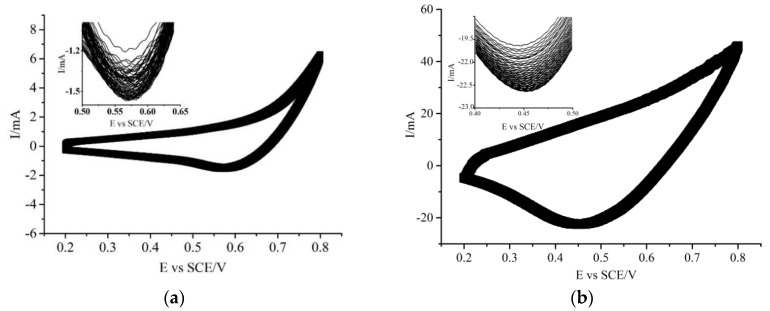
One hundred consecutive CV recorded at the CuNWE (**a**) and CuO/CuNWE (**b**) in a 0.075 M NaOH solution at a scan rate of 0.05 V s^−1^.

The CV curves of the CuNWE and CuO/CuNWE recorded in an organics-absent alkaline solution (0.075 M NaOH) over a potential range of 0.2 to 0.8 V (vs. saturated calomel electrode, SCE) is presented in Figure 3. The responses of both the CuNWE and CuO/CuNWE during positive scan (0.2 to 0.8 V) and negative scan (0.8 to 0.2 V) are similar to results reported on the copper-based electrodes [28,31] where no anodic peak related to the oxidation of Cu(II) to Cu(III) is observed but a cathodic peak that can be attributed to the conversion of Cu(III) to Cu(II) appears [24,35]. Compared with the CuNWE, however, the CuO/CuNWE displays a substantially higher anodic current and cathodic peak current. This result is consistent with the SEM observation that the in situ growth of CuO nanowires on the surface of the porous CuNWE can lead to a rise in active surface area. As shown in Figure 4, compared to the CuNWE, no significant decrease in anodic current or cathodic current is found on the CuO/CuNWE during 100 consecutive scans, implying that the CuO/CuNWE is electrochemically stable in alkaline medium.

#### 2.2.2. Electrochemical Behavior in Organics-Present Alkaline Medium

**Figure 5:**
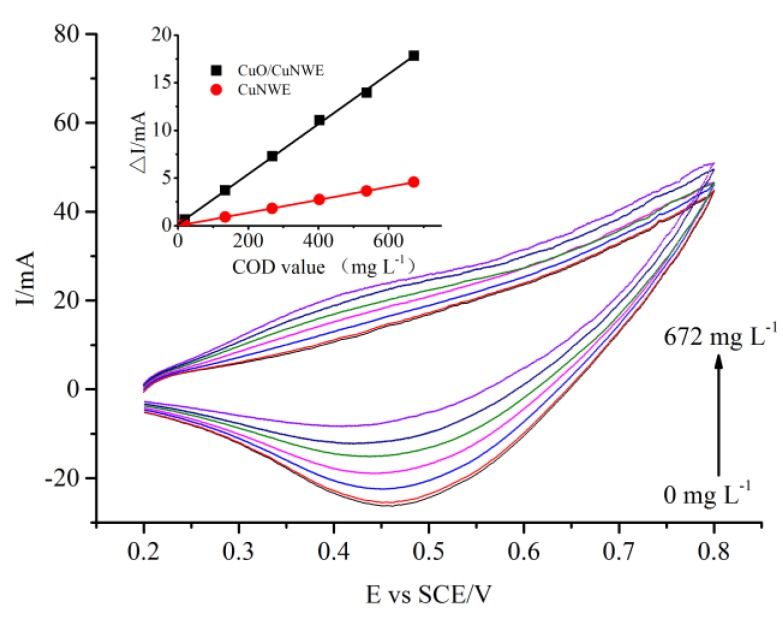
CV curves recorded at the CuO/CuNWE in a 0.075 M NaOH solution with various COD values (0 mg L^−1^, 19.2 mg L^−1^, 134.4 mg L^−1^, 268.8 mg L^−1^, 403.2mg L^−1^, 537.6mg L^−1^ and 672 mg L^−1^) at a sweep rate of 0.05 V s^−1^. The inset shows the linear relation between net change in cathodic peak current (ΔI) and COD value.

The electrocatalytic activity of the CuNWE and CuO/CuNWE in organics-present alkaline medium were also investigated by CV in synthetic aqueous samples with known COD values, where glucose was used as the standard COD test reagent to prepare the samples. As shown in Figure 5, in the positive scan the value of anodic current on the CuO/CuNWE increased with the COD value, suggesting a good response to the organic compound. During the negative scan, however, the peak current decreased with the COD value. This phenomenon is commonly attributed to the fact that the higher the COD value in the sample the lower the amount of Cu(III) species since Cu(III) species can be consumed by organics via Equation (5) [36]. The relation between the net change in cathodic peak current and the COD value can be fitted by a linear regression equation (see inset of Figure 2). A similar linear relationship can also be observed on the CuNWE (see inset of Figure 5). However, the slope of the linear regression line on the CuO/CuNWE is much higher than that on the CuNWE, further confirming that the growth of CuO nanowires on the CuNWE can greatly improve the performance of the electrode.

#### 2.2.3. Optimization of Scan Rate and Alkaline Concentration for COD Measurement Using the CuO/CuNWE

**Figure 6:**
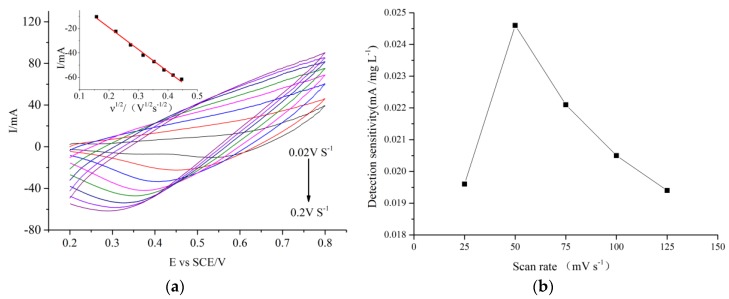
(**a**) CV curves recorded in 0.075 M NaOH solution containing 0.7 mM glucose at different scan rates. Inset is the relationship between peak current and scan rate. (**b**) effect of scan rate on the sensitivity to COD.

Figure 6a presents the CV curves recorded at the CuO/CuNWE in a 0.075 M NaOH solution containing 0.7 mM glucose (about 134.4 mg L^−1^ in COD) at a different scan rate. The inset of Figure 6a shows that the cathodic peak current is linearly related to the square root of the scan rate. The observed relationship between peak current and scan rate reveals that the electrocatalytic reaction is a typical diffusion-controlled process. To choose a suitable scan rate for COD measurement using the CuO/CuNWE, the effect of scan rate on the sensitivity to COD in the glucose samples with various COD values (0, 19.2, 134.4, 268.8, 403.2, 537.6 and 672 mg L^−1^) were investigated in a 0.075 M NaOH solution. It is clear from Figure 6b that a scan rate of 0.05 V s^−1^, which was also applied in other related works [19,28,35,36], is an optimum value for COD measurement. 

**Figure 7:**
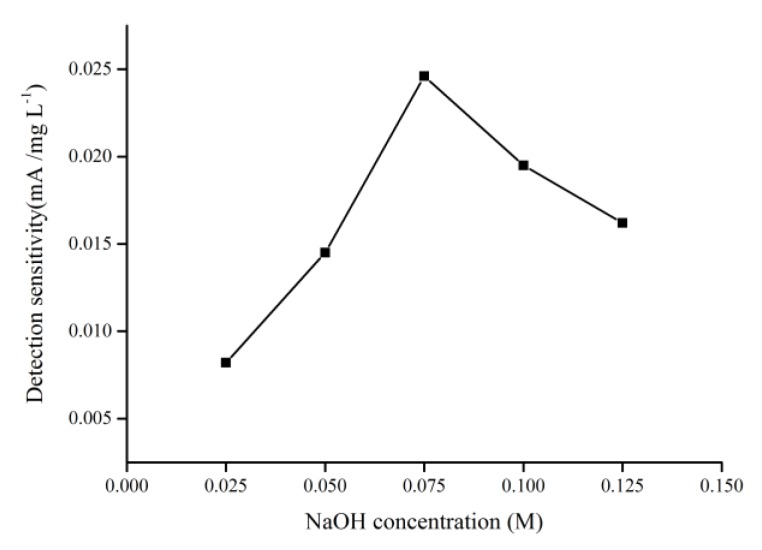
The correlation between sensitivity to COD on the CuO/CuNWE and NaOH concentration.

According to the mechanism presented in Equations (1)–(5), the NaOH solution can affect the production of Cu(III) species on the electrode surface. Therefore, the effects of NaOH concentration on the sensitivity to COD on the CuO/CuNWE in the glucose samples with various COD values (0, 19.2, 134.4, 268.8, 403.2, 537.6 and 672 mg L^−1^) were also investigated by CV at a sweep rate of 0.05 V s^−1^. As shown in Figure 7, the sensitivity of CuO/CuNWE initially increased with increasing NaOH concentration and then decreased as the NaOH concentration exceeded 0.075 M. The initial rise in sensitivity might be related to the fact that a high NaOH concentration would favor the formation of Cu(III) species on the electrode surface based on Equations (3) and (4) [24]. However, when the concentration of NaOH increased further, a high background noise could be observed with lots of bubbles appearing on the surface of the CuO/CuNWE, suggesting that water splitting became a dominant reaction [8] and thus resulted in a decline in sensitivity when the NaOH concentration was above 0.075 M. Similar experimental phenomena have also been reported by Felix et al. and Fan et al. [24,37]. Therefore, a NaOH concentration of 0.075 M is chosen for determination of COD using the CuO/CuNWE.

### 2.3. COD Measurement by CuO/CuNWE-Based Electrochemical Sensor

**Figure 8:**
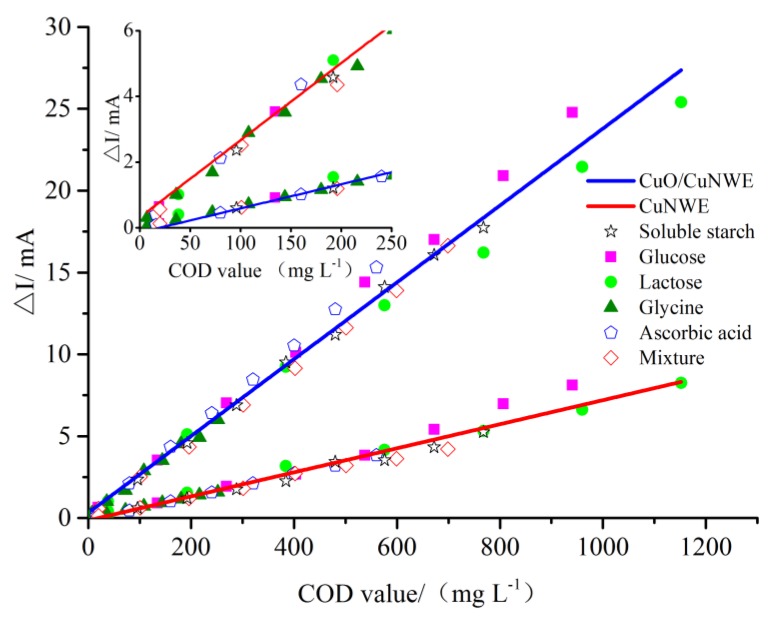
The net increase in cathodic peak current (ΔI) versus theoretical COD values of four organics (glucose, lactose, glycine and ascorbic acid), their mixtures, and soluble starch in a 0.075 M NaOH solution. Inset is the magnified view at low COD values.

To obtain the calibration curve for COD measurement by CV, four organics (glucose, lactose, glycine and ascorbic acid), their mixtures, and soluble starch with known theoretical COD values were selected as standard samples and the net change in cathodic peak current (ΔI) was plotted as a function of COD value. As can be seen in Figure 8, on both CuNWE and CuO/CuNWE a linear relationship between ΔI and COD value can be observed. From the slope of the linear regression line, we can obtain a sensitivity of ~0.0246 mA/(mg L^−1^) on the CuO/CuNWE and about 0.0068 mA/(mg L^−1^) on the CuNWE. The result indicates that, in comparison with the CuNWE, the CuO/CuNWE exhibit a much better performance for COD measurement. In addition, the detection limit (DL) of the proposed method using the CuO/CuNWE is determined to be about 2.3 mg L^−1^ by DL = 3σ/s [38], where σ and s are the standard deviation of the blank signal and the sensitivity, respectively. As can be seen from Table 1, the sensitivity of the CuO/CuNW-based COD sensor (namely ~2.46 × 10^−2^ mA/(mg L^−1^)) is much higher than those of the Cu-based COD sensors reported in the literature (ranging from 6.8 × 10^−9^ to 1.7×10^−3^ mA/(mg L^−1^)). In addition, in comparison with the reported Cu-based COD sensors with a detection limit of ~2 mg L^−1^ (except the NiCu-based sensor [12]), the COD sensor proposed in this work also possesses a wider linear range; namely, 5–1153 mg L^−1^.

**Figure 9:**
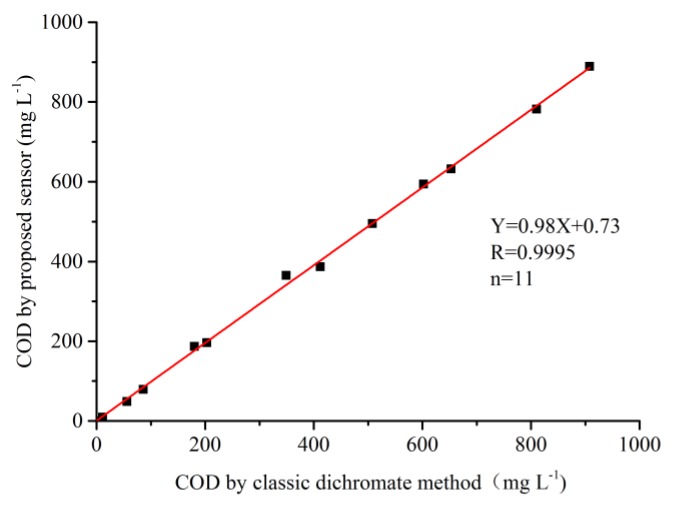
The relation between the COD values of synthetic samples (organic mixture) determined by the CuO/CuNWE-based electrochemical method and the classic dichromate method.

To make a comparison between the proposed electrochemical method using the CuO/CuNWE and the classic dichromate method, the correlation between two methods for measurement of COD in synthetic samples of different COD values obtained by mixing all four chemicals was also investigated, and the result is illustrated Figure 9. From Figure 9, we can observe a highly significant linear dependence between two methods (r = 0.9995, *p* < 0.01, n = 11). Moreover, the slope of the fitted line (0.98) is closed to unity, indicating that the COD values obtained by the CuO/CuNWE-based sensor is almost the same as those by the classic dichromate method.

The reproducibility of the CuO/CuNWE was evaluated using a synthetic aqueous sample prepared with glucose as the standard COD test reagent. The net increase in cathodic peak current ΔI from cyclic voltammograms were determined using a series of CuO/CuNWEs prepared in the same way and the relative standard deviation (RSD) of measurement of ΔI was found to be about 2.23% (n = 6). In addition, the CuO/CuNWE also showed good operational stability; namely, no significant performance change (RSD = 3.48%) was observed after the application of the same electrode for 3 assays every day for a whole week. For this test, when the CuO/CuNWE was not being used, the electrode was preserved in ultra-pure water at room temperature. To check the anti-interference ability of the CuO/CuNWE, the influence of chloride ion, a ubiquitous ion in most water systems, on the detection of COD was also investigated by CV. In the presence (1 M NaCl) or absence of chloride ion, the current response of the CuO/CuNWE electrode showed no apparent difference toward organic compound oxidation (19.8 mg L^−1^ COD in 100 mL NaOH 0.075 M solution). The result confirms that the CuO/CuNWE exhibits good resistance to surface poisoning of chloride ion.

In order to examine the effectiveness and feasibility of the CuO/CuNWE sensor in practical application, four real samples were collected and measured by the CuO/CuNWE-based electrochemical method. For comparison, these samples were also analyzed by the standard dichromate COD method. As shown in Table 2, the relative difference between the COD values in four samples measured by two methods are in the range of −6.67% to 2.31%. The good agreement between two methods suggests a great potential in the practical application of the CuO/CuNWE electrochemical sensor for COD measurement. 

## 3. Materials and Methods 

### 3.1. Reagents 

All chemicals were of analytical grade and used as received without further purification. Cu(OH)_2_, NaOH, ethylenediamine (EDA, >99.9%), N_2_H_4_·2H_2_O (80 wt%), and four organic compounds were used to prepare standard and synthetic samples with known COD values: glucose, ascorbic acid, lactose, and glycine, which were purchased from Aladdin Chemistry Co., Ltd. All solutions used in the studies were prepared with ultra-pure water (resistivity >18.3 MΩ·cm). Real water samples were collected from surface waters or wastewaters in Hangzhou (China). Water samples were stored in the dark at 4 °C and analyzed within 48 h. The temperature of the electrochemical cells was maintained at 25  °C for all experiments. 

### 3.2. Instrumentation 

Electrochemical measurements were performed on an electrochemical workstation (CH Instruments 660E, Shanghai Chenhua Co. Ltd., Shanghai, China) and a standard three electrodes system. The morphology of the samples was examined using a scanning electron microscope (SEM, Hitachi S-4700, Hitachi High-Technologies Corporation, Tokyo, Japan) operating at 15 kV. X-ray diffraction (XRD) patterns of the samples were obtained using a Rigaku D/max-RA powder diffraction-meter (Rigaku, Tokyo, Japan) equipped with Cu Ka radiation.

### 3.3. Preparation of CuO/CuNW Electrode

#### 3.3.1. Preparation of Cu Nanowires

Cu nanowires used in the experiments were synthesized via a cheap and environmentally friendly method developed by our group [27]. In a typical synthesizing procedure, 120 g sodium hydroxide was dissolved in 200 mL water. After cooling down to room temperature, the solution was transferred into a 500 mL three-necked flask followed by the addition of 0.098 g of Cu(OH)_2_, 2.5 mL of EDA and 0.2 mL of hydrazine solution (35 wt%) sequentially. The obtained mixture was placed in a 70 °C waterbath for 1 h. The reddish end product (CuNWs) was washed and collected by centrifugation–redispersion in pure water and ethanol, respectively, to remove inorganic and soluble compounds, as well as residual EDA and N_2_H_4_. 

#### 3.3.2. Preparation of Porous CuNWE

**Figure 10:**
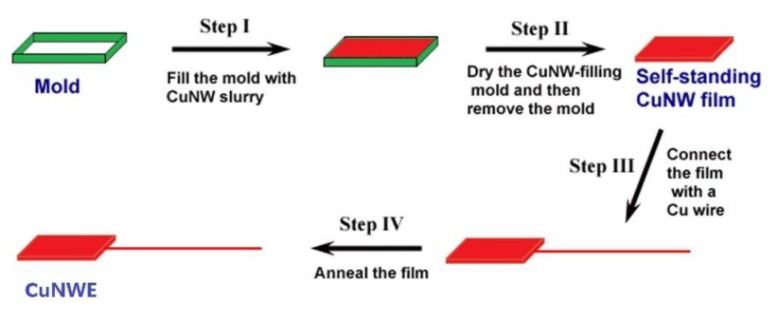
Schematic illustration for preparation of CuNWE.

The preparation procedure of the CuNWE was similar to that reported in our previous work [26]. Briefly, one third of the above-obtained CuNW slurry was filled into a mold with a size of 0.4 × 0.5 × 0.05 cm^3^ and then squeezed the excess ethanol out to ensure the structural stability. After being dried in N_2_ atmosphere at room temperature, the mold was removed and the obtained CuNW film was connected with a Cu wire (~1.5 mm in diameter). Then, the CuNW film was annealed in Ar atmosphere at 600 °C for 30 min to get the CuNWE. The scheme for preparation of the CuNWE is shown in Figure 10.

#### 3.3.3. Preparation of CuO/CuNWE

**Figure 11:**
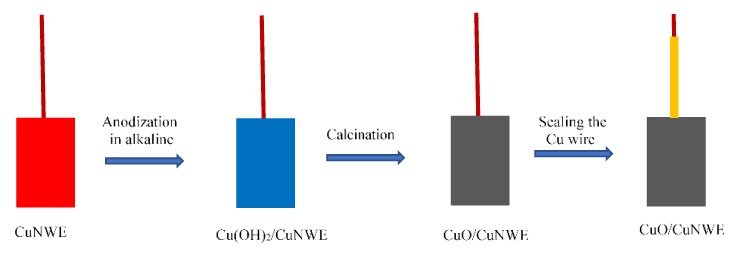
Schematic illustration for preparation of CuO/CuNWE.

In order to obtain CuO/CuNWE, the as-prepared CuNWE was anodized in an alkali solution (3 M NaOH) for a very short period of time (namely ~1 min) under potentiostatic oxidation at a voltage of 1.3 V. The cathode was a Pt plate with a size of 1 cm × 1 cm, and the distance between the anode and the cathode was about 1.2 cm. After being anodized in an alkaline medium, the electrode was covered with blue materials; namely, Cu(OH)_2_ nanowires (the as-formed electrode was denoted as Cu(OH)_2_/CuNWE). In order to convert Cu(OH)_2_ into CuO, the anodized electrode was calcinated at 300 °C for 2 h in Ar atmosphere. Finally, the Cu wire, which was connected to the electrode, was sealed with epoxy resin to prevent it from contact with electrolyte solution during electrochemical measurements. The scheme for preparation of the CuO/CuNWE was shown in Figure 11.

### 3.4. Detection of COD

In order to testify the practical application of CuO/CuNW electrode, all samples were analyzed by both the proposed electrochemical method and the traditional COD chemical method [39], respectively. In this study, the electrochemical COD measurement was performed using an electrochemical workstation and a standard three electrodes system with CuO/CuNWE as the working electrode, a platinum wire as the counter electrode and a saturated calomel electrode (SCE) with saturated KCl as the reference electrode. The water sample was pretreated by mixing 100 mL water sample with 0.3 g NaOH (as the electrolyte) in the beaker and homogenizing with a magnetic stirrer. The cyclic voltammograms were recorded by cycling the potential between 0.2 and 0.8 V vs. SCE at a scan rate of 0.05 V s^−1^.

## 4. Conclusions

In this paper, a self-supported CuO/CuNWE was prepared by anodization of a self-supported porous CuNWE, which was obtained by thermal annealing of Cu nanowires, anodizing in alkaline medium, and subsequent calcination. The as-prepared electrode could be applied as a sensor for the determination of the COD value by cyclic voltammetry. Owing to the superior electrocatalytic activity, the CuO/CuNWE showed extremely high sensitivity for COD detection. Under optimized alkaline concentration and scan rate, the developed sensor exhibited a linear range of 5 to 1153 mg L^-1^ and a detection limit of 2.3 mg L^-1^. Meanwhile, the proposed electrochemical method based on the CuO/CuNWE had shown a very good agreement with the classic dichromate method for COD measurement in both the synthetic and real water samples.

## Figures and Tables

**Table 1:** Comparison between the reported Cu-based sensors and the one described in this work.

Electrochemical Sensors Based on Different Electrodes	Linearity (mg L^−1^)	DL (mg L^−1^)	Sensitivity (mA /(mg L^−1^))	Reference
Cu/CuO	53.0–2801.4	20.3	4.717 × 10^−4^	[7]
nano-Cu/Cu	4.8–600	3.6	4.538 × 10^−4^	[8]
nano-Cu/ GCE	15–629.3	1.7	1.7 × 10^−3^	[9]
nano-Cu/ Cu cable	2–595	2.6	1.9 × 10^−4^	[10]
CuO nafion /Cu	50–1000	2.11	8.3 × 10^−6^	[11]
NiCu	10–1533	1.0	1.41 × 10^−4^	[12]
CuO/AgO	53–394	28	6.8 × 10^−9^	[17]
Cu-Co/Au	1.92–768	0.609	8.88 × 10^−4^	[18]
CuO/CuNWE	5–1153	2.3	2.46 × 10^−2^	This work

**Table 2:** Measurement results for real water samples by CuO/CuNWE-based electrochemical method and standard dichromate method.

Samples	Conventional Methods	Proposed Method RSD(%)		Relative Difference(%)
	COD_cr_ (mg L^−1^)	COD (mg L^−1^)	n = 5	
The West Lake, Hangzhou	12	11.2 ± 0.6	5.36	−6.67
The Shangtang river, Hangzhou	26	24.3 ± 1.1	4.52	−6.54
Papermaking wastewater	243	248.6 ± 11.1	4.47	2.31
Pharmaceutical wastewater	529	519.1 ± 14.8	2.86	−1.87

## Supplementary Materials

The following are available online, Figure S1: SEM images of the morphology of CuNWE after anodized at different voltages for 5 min in a 3M NaOH solution, Table S1: Effect of the applied voltage on the anodization of CuNWE.
