# Supplementary material for: A Self-Supported CuO/Cu Nanowire Electrode as Highly Efficient Sensor for COD Measurement
Source: Molecules. 2019 Aug 28;24(17):3132. doi: 10.3390/molecules24173132 (PMC6749378; doi:10.3390/molecules24173132)
Supplement: Supplementary file 1 [file molecules-24-03132-s001.pdf]

# A self-supported CuO/Cu nanowire electrode as highly efficient sensor for COD measurement

**Xinwen Huang[1], Yingying Zhu[1], Wanquan Yang[1,2], Anhua Jiang[1], Xiaoqiang Jin[1], Yirong Zhang[1], Liang Yan[2], Geshan Zhang[3], and Zongjian Liu[3,*]**

[1] Institute of Environment, College of Chemical Engineering, Zhejiang University of Technology, Hangzhou, 300014, P.R. China
[2] Powerchina Huadong Engineering Corporation Limited, Hangzhou, 311122, P.R. China
[3] College of Chemical Engineering, Zhejiang University of Technology, Hangzhou, 300014, P.R. China

Table 1S Effect of the applied voltage on the anodization of CuNWE

| Entry No. | Voltage applied (V) | NaOH concentration (M) | Anodization duration (min) | The color of the anode observed |
|---|---|---|---|---|
| 1 | 1.1 | 3 | 5 | No change |
| 2 | 1.3 | 3 | 5 | Change from reddish brown to blue |
| 3 | 1.5 | 3 | 5 | Change from reddish brown to black dotted with blue |
| 4 | 2.0 | 3 | 5 | Change from reddish brown to black |

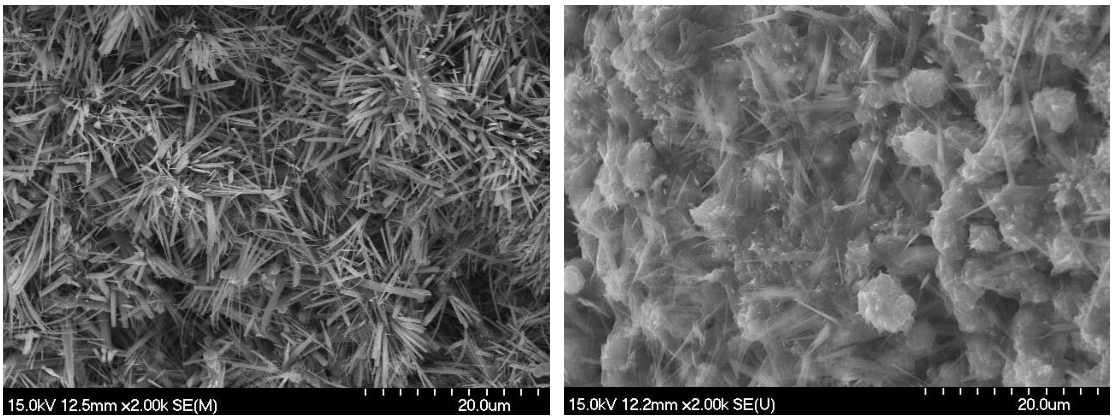


(a) (b)

**Fig. 1S** SEM images of the morphology of CuNWE after anodized at different voltages for 5 min in a 3M NaOH solution: (a) 1.3 V and (b) 2.0 V.
